# A Series of COVID-19 Cases With Findings in the Gastrointestinal and Hepatobiliary System

**DOI:** 10.7759/cureus.22602

**Published:** 2022-02-25

**Authors:** Dongling Wu, Sean Hacking, Lili Lee

**Affiliations:** 1 Pathology and Laboratory Medicine, Northwell Health, Northshore Long Island Jewish Hospital, Manhasset, USA; 2 Pathology and Laboratory Medicine, Warren Alpert Medical School of Brown University, Providence, USA; 3 Pathology and Laboratory Medicine, New York University Long Island School of Medicine, Long Island, USA

**Keywords:** case report series, case series, atypical covid-19 symptoms, microthrombi, gastrointestinal infection, covid-19

## Abstract

Coronavirus disease 2019 (COVID-19) caused by severe acute respiratory syndrome coronavirus 2 (SARS-CoV-2) has rapidly spread worldwide. Most of the infected patients present with respiratory symptoms and acute lung damage. Here, we present three cases of patients with COVID-19 disease whose main clinical manifestations are gastrointestinal symptoms. In our first case, we present a COVID-19 patient with histologic findings associated with ischemic necrosis of the small bowel. In the second and third cases, we demonstrate acute cholecystitis and histology showing microvascular thrombosis. These three cases highlight the ischemic and thrombotic changes seen in the setting of COVID-19 infection without classic respiratory symptoms, with resulting severe gastrointestinal and hepatobiliary disease requiring surgical management. Although the bile or stool viral load was not tested in these patients, the small intestine and gallbladder were infected with SARS-CoV-2, most likely via the epithelial angiotensin-converting enzyme 2 (ACE2) receptor.

## Introduction

Severe acute respiratory syndrome-associated coronavirus-2 (SARS-CoV-2) is a novel virus that belongs to the coronavirus family, causing coronavirus disease-2019 (COVID-19) [1], a disease first discovered in Wuhan, China in December 2019 which quickly led to an ongoing pandemic. As of today, millions of people have been infected with this virus in the United States. Most infected patients present with a fever, cough, and other respiratory symptoms. Approximately 25% of infected patients have gastrointestinal symptoms including nausea, vomiting, and diarrhea, with up to 39% of patients having elevated alanine aminotransferase (ALT) and aspartate aminotransferase (AST) levels [2]. However, a few cases of COVID-19 present as acute cholecystitis. Here, we summarize findings from the gastrointestinal and hepatobiliary systems of three SARS-CoV-2 infected patients, two of whom presented with acute calculous cholecystitis. The goal of this case study is to present the clinical and histologic features of infected patients with severe gastrointestinal and hepatobiliary disease manifestations.

## Case presentation

Case 1

A 69-year-old male with a past medical history of chronic kidney disease status post-renal transplant, coronary artery disease, congestive heart failure, type II diabetes mellitus, and recent COVID-19 testing was positive one month prior to presentation. He presented to the hospital with severe chest and abdominal pain and was found to have a non-ST elevated myocardial infarction (NSTEMI), ischemic bowel and portal vein thrombosis, all status post-heparin therapy. Cardiac catheterization with contrast did not reveal obstruction. The CT scan showed pneumatosis of the distal ileum, including mesenteric venous air and portal venous thrombus, which was non-occlusive. The patient underwent a diagnostic laparotomy and right hemicolectomy.

Gross examination of the right hemicolectomy showed a 75.0 cm in length × 5.0 cm in circumference segment of the small bowel and colon. A 9.5 cm × 0.5 cm diameter appendix is present, and the ileum measured 3.5 cm in thickness. No mesentery was identified in the colon.

The ileal serosa was gray-tan, glistening, and diffusely covered by tan-gray exudates. The ileal mucosal surface showed a macroscopic ischemic-appearing area. The cecal serosa was focally exudative, while the mucosa was purple in color.

**Figure 1:**
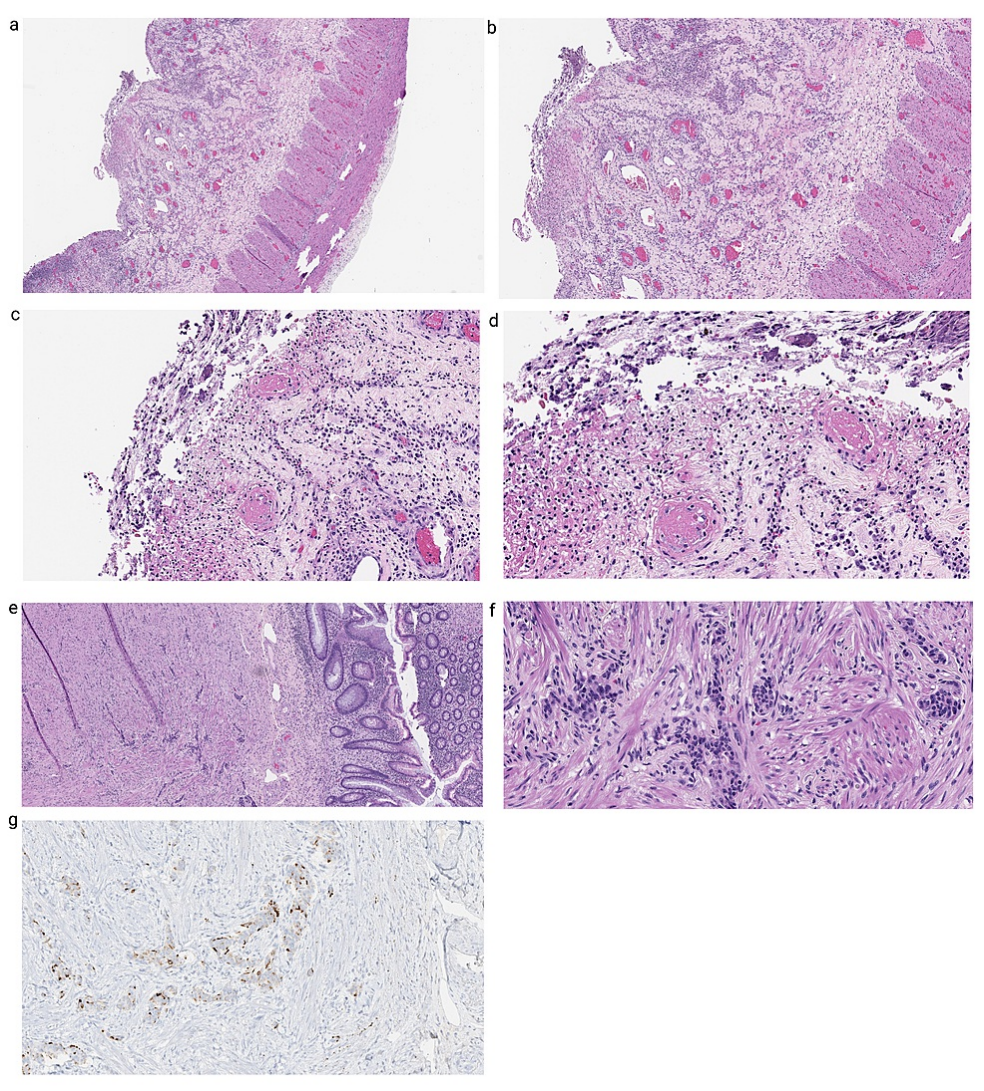
Case 1: small bowel resection (a) 4× and (b) 10× demonstrate mucosal ulceration, fibrinopurulent exudates, and mural congestion; (c) and (d) 20× demonstrate vascular microthrombi; (e) 4× and (f) 20× incidental neuroendocrine tumor; (g) chromogranin stain

Histological examination of the ileum, appendix, and right colon revealed ischemic enteritis featuring active ileitis, mucosal ulceration, fibrinopurulent exudates, and foci of complete transmural necrosis (Figure 1a, 1b). Mesenteric vascular thrombi were also seen (Figure 1c, 1d). Early ischemic change with active enteritis involving the proximal resection margin was present, while the distal resection margin was viable. There were three reactive lymph nodes associated with the resection, and serosal fibrous adhesions were also seen. An incidental well-differentiated neuroendocrine tumor measuring 0.5 × 0.4 × 0.4 cm^3^ was found in the appendiceal tip; 15.0 cm and 80.0 cm from the nearest distal and proximal margin, respectively. The mitotic rate in the most mitotically active area was 0/2 per mm^2^. Nuclei demonstrated monomorphic cells with eosinophil cytoplasm, minimal atypia, and salt and pepper chromatin condensation (Figure 1e, 1f). Immunohistochemical staining for chromogranin was positive (Figure 1g).

Case 2

A 26-year-old female was admitted to the obstetric service for vaginal delivery at 37 weeks of gestation and was found to be infected with the COVID-19 virus, confirmed by PCR. Two days after an uncomplicated vaginal delivery, she presented with acute onset right upper quadrant abdominal pain. Laboratory results were significant for a slightly elevated alkaline phosphatase (ALP) at 130, with normal AST: 30 (U/L) and ALT: 19 (U/L). A CT scan of the abdomen showed a large, distended gallbladder with multiple stones, and a thickened wall measuring up to 4 mm and mild pericholecystic fluid concerning acute cholecystitis.

The patient was admitted for laparoscopic cholecystectomy. On inspection, the gallbladder was edematous, consistent with acute cholecystitis. Postoperative gross examination revealed an intact gallbladder measuring 12.5 × 4.0 × 3.2 cm^3^. The cystic duct was patent, measuring 0.9 cm in length and 0.8 cm in diameter. The serosa was tan-purple and smooth, and the hepatic surface was scabrous. Bile was present in the lumen and was green-brown and tenacious in nature. Multiple calculi, ranging from 0.5 to 1.5 cm, were found showing a tan-yellow and smooth cut surface.

**Figure 2:**
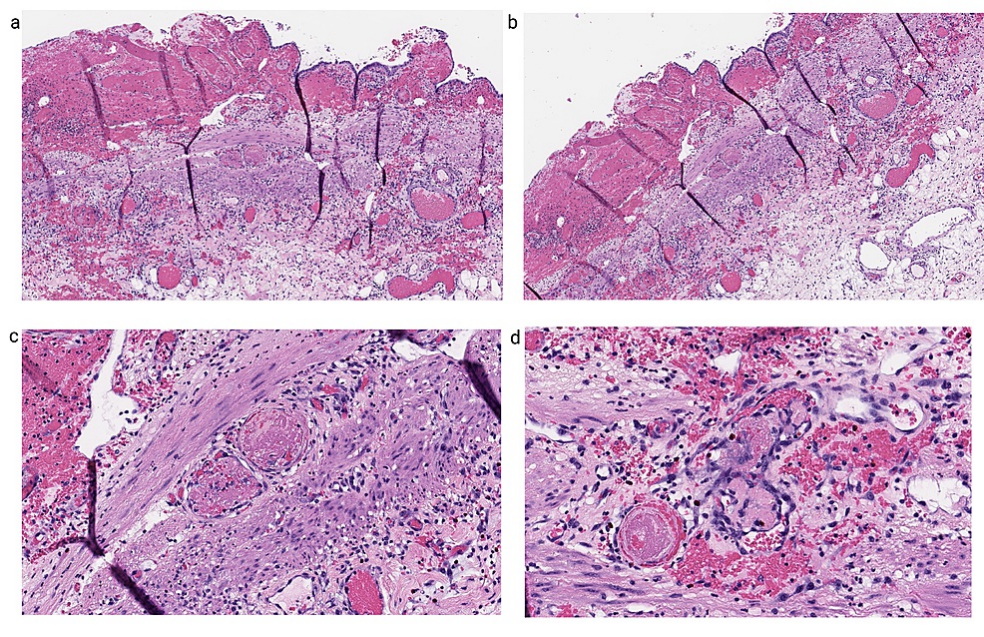
Case 2: cholecystectomy (a) 4× and (b) 10× demonstrate acute cholecystitis with ulceration and mural congestion; (c) and (d) 20× demonstrate vascular microthrombi.

Histologic examination showed features of transmural acute inflammation infiltrates indicating cholecystitis with mucosal ulceration and fibrinopurulent exudates (Figure 2a, 2b), areas of complete transmural necrosis, and multiple foci of submucosal vascular thrombi (Figure 2c, 2d).

Case 3

A 72-year-old female with a past medical history significant for Churg-Strauss syndrome, hereditary hemorrhagic telangiectasia with epistaxis, and small bowel angioectasias was initially hospitalized for SARS-CoV-2 infection, which was complicated by an acute arteriovenous fistula with anemia requiring multiple transfusions. During hospitalization, she developed right upper quadrant abdominal pain. A CT scan showed acute cholecystitis and a 1.8 cm stone. The patient had a percutaneous cholecystostomy for symptomatic control and was scheduled for a laparoscopic cholecystectomy after she recovered from active COVID-19 disease.

Gross examination of the resection specimen revealed a shaggy, previously disrupted gallbladder measuring 9.0 × 4.6 × 2.5 cm^3^. The cystic duct was grossly recognizable and patent, measuring 0.5 cm in length and 0.2 cm in diameter. The serosa was tan-red, and the hepatic surface was tan-brown and shaggy. The mucosa was tan-brown, shaggy, and granular. The lumen contained innumerable indurated to friable choleliths measuring in aggregate 4.5 × 3.5 × 1.0 cm^3^, the largest of which measured 1.5 × 1.0 × 0.7 cm^3^.

**Figure 3:**
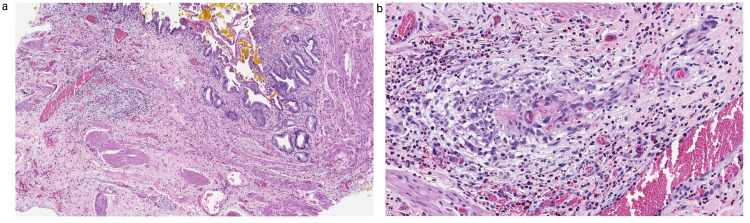
Case 3: cholecystectomy (a) 4× demonstrates acute and chronic cholecystitis; (b) 20× demonstrates vascular microthrombi.

Histologic examination shows neutrophil-mediated mucosal injury and expansion of the lamina propria by a mixed inflammatory cell infiltrate. Additionally, foci of submucosal vascular thrombi were identified (Figure 3a-3d). Vascular congestion and areas of hemorrhage were noted in the submucosa. No evidence of transmural necrosis or vasculitis was seen.

## Discussion

COVID-19 is a new multisystem infectious disease caused by the novel coronavirus, SARS-CoV-2. The most common presentation of this disease is severe pneumonia, which can rapidly progress to acute respiratory distress syndrome (ARDS) [3]. The mortality of this disease is approximately 0.25%, with the majority of cases progressing to diffuse alveolar damage [4]. Studies have also revealed that COVID-19 infection can cause a hypercoagulative environment resulting in microvascular thrombi in multiple organ systems [5]. Magro et al. reported a case series of COVID-19 lung tissue characterized by fibrin microthrombi [6]. Falasca et al. reported a series of 22 autopsies on decedents with severe COVID-19 infection and demonstrated prominent microvascular damage and thrombosis [7]. One explanation for vascular thrombi in the background of COVID-19 could be related to the angiotensin-converting enzyme 2 (ACE2) receptor and cytokine storms [8,9]. The binding and activation of the ACE2 receptor will cause damage to endothelial cells. The disruption of the ACE2 receptor by the virus then causes an imbalance in the ACE2 axis, which induces the cytokine IL-6, TNF-a storm, and other inflammatory responses [8,9]. Subsequentially, this activates the coagulation cascade and the platelet response, which eventually induces thrombotic effects throughout the body [10]. Ni et al. reported that SARS-CoV-2 attaches to cells via the ACE2 entry receptor, which is widely expressed on endothelial cells [10].

The presentation of COVID-19 infection is variable, with up to 20% of infected patients presenting with gastrointestinal symptoms, including nausea, vomiting, diarrhea, abdominal pain, and even GI bleeding. One meta-analysis study from Parasa et al. reviewed 29 studies with a total of more than 4000 patients, finding 7.4% of patients presented with diarrhea, and 4.6% with nausea and vomiting [11]. Buckholz et al. reported a rare case of COVID-19 infected patients presenting with gastrointestinal bleeding. The endoscopic findings showed mucosa edema and bleeding. Histology showed hemorrhage in the lamina propria and fibrin microthrombi [12]. Yantiss et al. first described a three-case cohort of SARS-CoV-2 infection-induced small intestine and colon changes. The infected epithelial cells show a "tufted appearance," a crowding of crypts with virus-related cytologic changes. The mucosa in reported cases also showed vascular thrombi [13].

Zhang et al. first postulated that ACE2 receptors are widely expressed in the small intestinal epithelium and that they may play an important role in SARS-CoV-2 transmission and evoking gastrointestinal inflammatory responses and gastrointestinal symptoms with ACE2 receptors [14].

Our paper presented gastrointestinal manifestations in a series of patients infected with SARS-CoV-2. One common feature of our case series was that these patients presented with mild gastrointestinal symptoms without any respiratory manifestations. We specifically demonstrated two cases of SARS-CoV-2 infection related cholecystitis. Both patients had no underlying gallbladder disease and presented with acute calculous cholecystitis without respiratory symptoms. The histology for both cases showed vascular thrombi, consistent with findings of systemic hypercoagulability described by numerous studies. Previously, a couple of papers have been reported showing acute cholecystitis as a complication of severe COVID-19 [15-19]. Three of these cases have histologic findings compatible with ours, further supporting that microthrombi are a common COVID-19-related pathologic change. Additionally, all six cases previously reported were ICU patients with severe acute pneumonia, and two developed sepsis that required prolonged hospitalization [19,20]. Our case series showed that acute cholecystitis can occur as an isolated clinical manifestation of SARS-CoV-2 infection without any respiratory symptoms. Even in patients with mild symptoms like we presented, microthrombi was still the most common finding. Zong et al. and Hong et al. showed ACE2 receptors are also highly expressed in gallbladder epithelium [19-21], making the gallbladder a prime target for the SARS-CoV-2 virus [21,22].

Case 2 was managed with a laparoscopic cholecystectomy after a CT scan confirmed the diagnosis. Case 3 underwent percutaneous transhepatic gallbladder drainage first for symptom control and then had an elective cholecystectomy after testing negative for the SARS-CoV-2 virus by PCR. Both patients fully recovered from symptoms of acute cholecystitis and SARS-CoV-2 infection without any complications. Currently, there is no consensus for the management of COVID-19-related acute cholecystitis. However, the Society of American Gastrointestinal and Endoscopic Surgeons (SAGES) has recommended that cholecystectomy remains the gold standard for treatment of acute cholecystitis in COVID-19 patients [23,24].

The finding of ischemic enteritis was interesting in our first case and consistent with multiple case reports which demonstrated the effects of hypoxia seen in the setting of SARS-CoV-2 infection. The ischemic bowel may be a consequence of compromised microvascular blood flow to the intestinal wall coupled with inflammatory activation, which can occur with or without pulmonary involvement [25]. Mesenteric vasculitis is commonly found in SARS-CoV-2 infection. A previous case report demonstrated multisystem inflammatory syndrome in children (MIS-C) caused by COVID-19 infection, where the patient’s presentation mimicked acute appendicitis [26]. Histologically, acute vasculitis was found in the mesentery with abundant acute inflammatory cells infiltrating artery walls. For some patients, it is possible that GI findings could be the initial COVID-19 manifestations, antedating respiratory symptoms.

Another interesting finding in our case series is the incidental low-grade neuroendocrine tumor. One large study of 1590 COVID-19 patients found that 18 (1%) cases had an incidental finding of cancer, which is higher than the incidence of cancer nationwide [27]. Another report from one single cancer institution estimated the mortality rate of SARS-CoV-2 in patients with cancer was 0.79%, statistically higher than in the non-cancer population [28]. With the pandemic affecting the capacity of hospitals, many studies have called for attention for the management of cancer patients to be patient-centered and dynamic [29].

## Conclusions

The manifestations of COVID-19 disease in the gastrointestinal and hepatobiliary systems can be clinically and pathologically diverse. As seen here, gastrointestinal manifestations of COVID-19 disease can be found in the setting of other pathologies. Determining the presence of COVID-19 and its relationship to a patient’s clinical course will be important for making diagnoses and determining subsequent therapeutic strategies. This is best performed with multiple clinical modalities and in collaboration with appropriate consultant subspecialties.
